# Use of Intersectionality Theory in Interventional Health Research in High-Income Countries: A Scoping Review

**DOI:** 10.3390/ijerph20146370

**Published:** 2023-07-15

**Authors:** Laura Tinner, Daniel Holman, Stephanie Ejegi-Memeh, Anthony A. Laverty

**Affiliations:** 1 Population Health Sciences, Bristol Medical School, University of Bristol, Bristol BS8 1UD, UK; 2 Department of Sociological Studies, The University of Sheffield, The Wave, 2 Whitham Road, Sheffield S10 2AH, UK; 3 Public Health Policy Evaluation, School of Public Health, Imperial College London, London SW7 2BX, UK

**Keywords:** intersectionality, health inequalities, health interventions, evidence synthesis

## Abstract

Background: Intersectionality theory posits that considering a single axis of inequality is limited and that considering (dis)advantage on multiple axes simultaneously is needed. The extent to which intersectionality has been used within interventional health research has not been systematically examined. This scoping review aimed to map out the use of intersectionality. It explores the use of intersectionality when designing and implementing public health interventions, or when analysing the impact of these interventions. Methods: We undertook systematic searches of Medline and Scopus from inception through June 2021, with key search terms including “intersectionality”, “interventions” and “public health”. References were screened and those using intersectionality and primary data from high-income countries were included and relevant data synthesised. Results: After screening 2108 studies, we included 12 studies. Six studies were qualitative and focused on alcohol and substance abuse (two studies), mental health (two studies), general health promotion (one study) and housing interventions (one study). The three quantitative studies examined mental health (two studies) and smoking cessation (one study), while the three mixed-method studies examined mental health (two studies) and sexual exploitation (one study). Intersectionality was used primarily to analyse intervention effects (eight studies), but also for intervention design (three studies), and one study used it for both design and analysis. Ethnicity and gender were the most commonly included axes of inequality (11 studies), followed by socio-economic position (10 studies). Four studies included consideration of LGBTQ+ and only one considered physical disability. Intersectional frameworks were used by studies to formulate specific questions and assess differences in outcomes by intersectional markers of identity. Analytical studies also recommended intersectionality approaches to improve future treatments and to structure interventions to focus on power and structural dynamics. Conclusions: Intersectionality theory is not yet commonly used in interventional health research, in either design or analysis. Conditions such as mental health have more studies using intersectionality, while studies considering LGBTQ+ and physical disability as axes of inequality are particularly sparse. The lack of studies in our review suggests that theoretical and methodological advancements need to be made in order to increase engagement with intersectionality in interventional health.

## 1. Introduction

The Marmot Review 2020 found that there has not been the expected reduction in health inequalities in the UK and some inequalities have widened [1]. This picture is similar across many other high-income countries, with strategies having limited success in dealing with the long term consequences of the financial crisis of 2008, among other structural issues [2].

These difficulties have given rise to new forms of framing health inequalities and an increased desire for strategies to overcome identified barriers and help to theorise and communicate their nature, causes and solutions [3]. Intersectionality explicitly considers multiple axes which may give rise to health inequalities and represents a potentially promising way forward on these issues [4]. The concept was first developed by Crenshaw in 1980 and is rooted in Black feminist and critical legal theory [5]. It is based on the premise that there are multiple social forces, social identities and ideological instruments through which power and disadvantage are expressed and legitimized [5]. While there is no clear consensus about the exact definition of intersectionality, a key focus on social justice and mutually cross-cutting and interacting dimensions of identity are commonly used [6,7]. Core principles include: (1) an explicit focus on structural factors or social determinants of health; (2) consideration of discrimination, particularly across more than one axis of inequality; (3) a focus on an equitable power dynamic with communities and users of services [8].

Intersectionality has been adopted in research to consider and assess ‘intersections’ of a wide range of identities and positions, including ethnicity, socioeconomic position, gender, LGBTQI+ and physical disability [9]. Intersectionality has been described as an essential theoretical framework that may be particularly useful to address health issues and their impact on the most vulnerable populations [6]. There are a range of analytic and theoretical frameworks that come under the concept of intersectionality, with substantial heterogeneity between these. Prominent examples such as the Intersectionality-Based Policy Analysis (IBPA) attempt to set out policy-based frameworks that support the decision-making processes among stakeholders working in health-related sectors [7,10]. This framework has provided a structure to critically analyse policy, to understand policy contexts and to generate innovative equity-focused policy solutions. Other frameworks aim to offer a context for conceptualising and interpreting intersectionality at the individual level for both quantitative and qualitative research [11]. However, despite the strong theoretical rationale for adopting intersectionality within public health research and a suite of developed frameworks for researchers to employ it, little is known about whether intersectionality (either these noted frameworks or other manifestations) is being translated into health-related interventional research. Mapping out the extent of intersectionality’s use within the field will help ascertain whether researchers are adopting this potentially useful concept into their applied research, or whether intersectionality may remain stagnant as a purely theoretical concept. Scoping out the current evidence base will also identify successful (or unsuccessful) adoption of intersectionality, to provide learning for other researchers developing or evaluating interventions. Until we have an idea of the extent of use, we are unable to make assessments of its interventional utility and make recommendations for implementation.

Within public health, the possibility of interventions exacerbating health inequalities has been highlighted and is beginning to be investigated through subgroup analyses [12,13]. However, interventions which focus on only one axis of inequality are similarly limited and risk worsening inequalities by not sufficiently tailoring delivery to different groups or evaluating the effects to account for interactions. Intersectionality could thus prove useful in designing and evaluating public health interventions, allowing researchers to determine the equitability of their intervention in a way that accounts for multiple interlocking inequalities, yet review evidence is lacking and the extent to which it is being employed is relatively unknown. One systematic review published in 2021 mapped the presence of intersectionality in quantitative health research [14]. However, this study did not explore the use of intersectionality in studies of health interventions, a gap we intend to address. Further, an overview by Heard et al. [15] provides some direction around how intersectionality is beginning to be incorporated into public health promotion and gives examples related specifically to public health interventions. For instance, consulting people from minority ethnic backgrounds at the start of intervention development to recognise alternative consequences for people who sit at unique social positions. Crucially for our review, Heard et al. reiterate that applying intersectionality to methodology remains under-explored, but there are innovative modelling methods emerging which may advance quantitative intersectionality-informed analysis [15,16].

Given this potential utility of intersectionality for interventional research, our scoping review seeks to clarify how intersectionality is being used within interventions through systematic scoping methods. It is hoped that, through doing this, we will gain a better idea of the landscape of interventions using intersectionality and can begin to identify successes, challenges and recommendations to help researchers design more equitable interventions. Our review provides an initial step in this process, through examining the extent to which intersectionality frameworks are used within interventional health research.

### Review Aim

Our review aim was: To explore what evidence is there on the use of theoretical and analytical intersectionality frameworks when designing and analysing public health interventions. Our scoping review set out to explore the use of intersectionality theory and/or frameworks when designing or implementing public health interventions. We also aimed to identify intersectionality-based analytical approaches to examining the impact of interventions on health inequalities. 

## 2. Methods

Given the nature of our aim, we used a scoping review to allow flexibility to capture a range of intersectionality frameworks. Scoping reviews summarise evidence to convey the breadth and depth of a field, and may involve some analytical reinterpretation of the research literature [17,18]. We followed the Preferred Reporting Items for Systematic reviews and Meta-Analysis extension for Scoping Reviews (PRISMA-ScR) reporting guidelines and align with recommended methods for scoping reviews [17,19,20,21].

We used a configurative approach to identify intersectionality frameworks supporting interventions. Configurative syntheses focus on gathering evidence to elucidate certain processes rather than testing any intervention effects. We thus aimed to organise available data to better understand and answer our review question.

### 2.1. Eligibility

We included studies exploring the utility of intersectionality frameworks either to design/implement or to analyse the impacts of public health interventions. We only included interventional health-related studies that aimed to effect physical or mental health outcomes. In line with the core concepts of intersectionality, our health inequality search terms included ethnicity, socioeconomic position, gender, LGBTQI+ and disability. We included studies which directly mentioned intersectionality theoretical or analytical frameworks within the paper.

#### 2.1.1. Inclusion Criteria 

To be included in our review, studies should address intersectionality when designing, implementing and/or evaluating interventions. We remained open to a range of uses of intersectionality, expecting heterogeneity between studies in both the degree to which it was employed as either a framework for design or analysis, as well as the way it was interpreted and applied. Therefore, there may be studies that mention that they use intersectionality as a lens in their early discussions during the design of the intervention as well as studies that employ intersectionality as a guiding framework throughout the whole of the design and evaluation process—and both these would be included under our criteria. All study types were accepted, including quantitative, qualitative and mixed-method approaches. 

We limited our inclusion criteria to high income countries for a few reasons. First, the intervention implementation context between high income countries (HICs) and low-and-middle income countries (LMICs) can be quite different, and reviews regularly separate inclusion criteria in this way. While intersectionality is a potentially useful concept for both contexts, the systems of power and oppression are likely different in different contexts, an idea beginning to be explored through applying intersectionality with the consideration of geographic and socioeconomic contexts [22]. One article highlights the second reason for reviewing HICs separate from LMICs, in that studies employing intersectionality in LMICs tend to be focused on immunization, HIV and violence and sexual abuse [23]—all public health topics quite different to health behaviour and mental health topics typical of HIC interventions. Finally, the recent systematic review on intersectionality in public health research cited in the background did include studies from both high and low-and-middle income countries and found that the vast majority of studies were conducted in high income countries [24]. Therefore, while our scoping review is limited by this omission, we do not expect there would have been many additional studies to include, yet a considerable amount more studies to screen.

To be included, we restricted studies to being health-related studies (i.e., measuring or analysing any indicator or topic related to physical or mental health of populations, as well as health system related outcomes), as opposed to educational or social outcomes, providing original results. 

#### 2.1.2. Exclusion Criteria

We excluded studies that were commentaries, editorials, book reviews or studies exclusively focused on educational, sociological or judicial issues. 

### 2.2. Search Strategy

The main search strategy was conducted in Medline and Scopus in June 2021. The search strategy was initially designed for Medline and then adapted to be replicated in Scopus. The search terms used were related to “intersectionality”, “interventions” and “public health” (Appendix A for full search strategy). The searches were not limited by year or language. In addition, the citations of identified key papers were screened to find relevant studies that had not been captured by the search strategy. The retrieved references were stored and managed using EndNote X9 (Clarivate, Emeryville/Berkley, CA, USA). References were exported to the web-based software Covidence (Veritas Health Innovation, Melbourne, Australia) for screening.

### 2.3. Screening 

The screening was conducted by two researchers (ARG and LT). Initially, the titles and abstracts of articles identified from the search strategy were screened against the inclusion and exclusion criteria. Then, the same process was carried out with the full text of selected articles. Any disagreement was solved through discussion with a third researcher (AAL). 

### 2.4. Data Extraction

A data extraction form was created including the following fields: study design, setting, number of participants, year of publication, country, target population, inequalities targeted, areas of intervention, summary of quantitative and qualitative outcomes, description of intersectionality framework, intersectionality approach and any additional notes.

### 2.5. Synthesis of the Results 

Research evidence from quantitative, qualitative and mixed-method studies was summarised narratively. We described studies according to their target populations and axes of inequality addressed. Studies were categorised into those where intersectionality frameworks were used (1) in order to design their interventions from the outset, and (2) after interventions were implemented in order to analyse effects.

### 2.6. Selection Process

**Figure 1:**
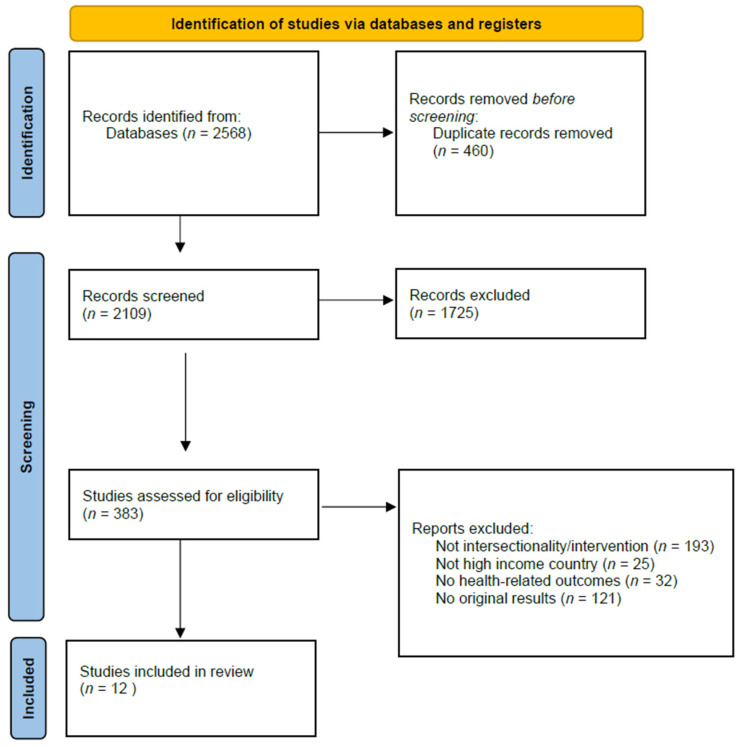
PRISMA Flow Diagram.

The search strategy retrieved 2568 references, of which 460 were duplicates (Figure 1). The titles and abstracts of 2108 references were screened, resulting in 383 references included in full text screening. The most common reasons for exclusion were “studies not using any intersectionality framework” (193 studies) and “studies not providing original results” (121 studies). Thirty-two studies were excluded as they did not contain any health-related outcomes and exclusively focused on educational, sociological and judicial outcomes. Twelve studies met the inclusion criteria and were included in the review.

## 3. Results 

### 3.1. Characteristics of Included Studies

Of the twelve included studies, six studies were based in the USA, four studies were conducted in the UK, one in Canada and one across several countries (USA, UK, Australia, New Zealand and Norway) (Table 1). All studies were published between 2014 and 2021. Six studies were qualitative and focused on alcohol and substance abuse (two studies), mental health (two studies), general health promotion (one study) and housing interventions (one study). 

The three quantitative studies examined mental health (two studies) and smoking cessation (one study), while the three mixed-method studies examined mental health (two studies) and sexual exploitation (one study). Full details of included studies are in Table A2. 

There was substantial heterogeneity in terms of the target populations. Included populations were: health researchers [25]; Polish migrants [26]; mental health service users [27]; women on (or previously on) opioid substitution treatment [28]; single adults in receipt of welfare for housing [29]; pregnant/postpartum women [30]; Asian men affected by mental illness [31]; people who smoke tobacco [32]; people receiving individual mental health counselling [33]; youth at risk of sexual exploitation [34]; homeless women [35]; Latina women who had experienced interpersonal violence [36]. The number of participants varied between 17 and 415, with lower sample sizes in the qualitative studies.

**Figure 2:**
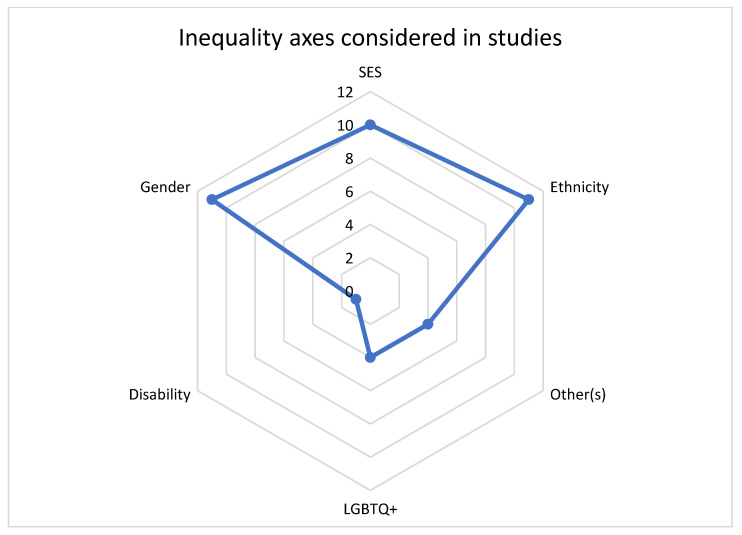
Axes of inequality considered in included studies.

The most commonly addressed individual axes of inequality were ethnicity and gender, which were considered by 11 studies each (Table 2 and Figure 2). SES was considered by 10 studies. LGBTQ+ was considered by four studies and disability by one study. Of our five pre-defined axes of inequality, only one study considered all five, with two studies considering four of these axes. Six out of the twelve studies considered three out of five of our pre-defined axes of inequality. There was heterogeneity regarding which intersections (e.g., sex X ethnicity X age), although SES, ethnicity and gender were considered by eight out of the twelve included studies. 

Eight studies used intersectionality to analyse the association between inequality axes and intervention outcomes [26,27,28,29,31,32,33,35]. The role of intersectionality within these studies was mainly related to the interpretation of the findings in terms of cross-cutting inequalities. Four of these studies analysed mental health related problems [27,31,33,35], one study focused on smoking cessation [32], one on housing [29], one on alcohol abuse and one on drug abuse [26,28]. There were considerable differences in the use of intersectionality. Whilst, in some studies, intersectionality clearly structured the analysis of the results [28,31,32], other papers only used intersectionality as one option for the interpretation of the findings [27].

Three studies utilised intersectionality to design interventions with an aim to enhance their effectiveness on reducing health inequalities [25,34,36]. In these studies, intersectionality was consistently used to guide the design of the interventions across the studies. One study used intersectionality for both the design of the intervention as well as the interpretation of findings [30] (Table 2). 

#### 3.1.1. Use of Intersectionality to Analyse Impacts of Interventions (Eight Studies)

Four of the studies which used intersectionality to analyse the effects of interventions were on mental health. These studies examined various intersections using gender, ethnicity, SES and LGBTQ+ indicators. In addition to analysis of intervention effects, one study used intersectionality to decide what axis of inequality should be used to measure effects [33]. The other three studies concluded their analyses by proposing intersectionality as a useful framework for improving or tailoring services in the future. These studies demonstrate an awareness and use of the key tenets of intersectionality: three of them use such frameworks from the outset, including Kivlighan et al. [33], which addresses the question of whether intersecting identities effect treatment outcomes. All of these studies conclude that the use of intersectional frameworks is beneficial in maintaining suitable awareness of social dynamics and recommend their further use. In one study by Lloyd et al. [27], issues of intersectionality were raised by the participants as a way to improve treatments. 

Of these studies on mental health, Lloyd et al. [27] interviewed and surveyed LGBQ+ patients who had completed a cognitive behavioural programme to overcome mental health issues specifically designed for sexual minorities. An important suggestion for developing and improving this therapy from the patients was the use of an intersectional lens to acknowledge the totality of people’s experiences and to move beyond a predominant focus on male sexuality and identity. Kivlighan et al. [33] used an intersectionality framework to investigate therapist–client interaction in relation to gender and ethnicity. Their analysis of 415 clients treated by 16 therapists found that there were differences in therapists’ ability to reduce psychological distress depending on the intersection of ethnicity and gender. Morrow et al. [31] used intersectionality as an analytical framework to explore Asian men’s diverse experiences of stigma and mental health, including how this is mediated by a range of other identities and experiences of racism, inequality and immigration. They report that participants understood and experienced their stigma as linked to their social identities and that intersectional frameworks must feature prominently in attempts to reduce mental health stigma. David et al. [35] used an intersectional framework in their mixed-method evaluation of mental health services delivery for women who are homeless. Their findings highlight the importance of tailoring treatments to account for the multiple forms of oppression faced by these women and assuming a culturally sensitive therapeutic stance in treatment. They specifically reference the intersectional oppression faced by these homeless women and advocate the use of an intersectional perspective in providing treatment.

The remaining four studies using intersectionality to analyse the impacts of interventions targeted smoking cessation, housing and alcohol and drug misuse. These studies all used intersectionality frameworks to assess whether the interplay of social identities is more important to consider than individual axes of inequality. Three of these studies examined whether outcomes differed according to intersectional markers of identity, and one recommended the consideration of intersectionality as a method to improve services. They did not, however, contain explicit reference to power dynamics or discrimination. 

Potter et al. [32] used an intersectional framework to assess how the interplay of multiple marginalised attributes contributes to smoking cessation. This study found that low household income was related to continued smoking but identified no interaction between marginalised attributes and relapse. Gleeson et al. [26] explored associations between social attitudes towards gender and access to services related to migrant status and social class according. Their findings suggested that Polish female migrants accessing alcohol-related services face barriers including social stigma and sexist attitudes towards women, and that these interact to create negative outcomes. Medina-Perucha et al. [28] studied the intersection of different types of inequalities in women receiving opioid substitution treatment with a focus on issues of stigma and discrimination. They highlighted that stigma interacts with other aspects of the women’s identities, including drug use and homelessness, to create negative outcomes. Wilkinson and Ortega-Alcázar [29] studied the impact of shared housing on young people and their wellbeing using an intersectional perspective. Their findings stressed the importance of considering the intersection of different types of inequalities on physical safety and violence, mental health and isolation.

#### 3.1.2. Intersectionality as a Tool to Design Interventions (Four Studies)

Three studies used intersectionality to design interventions [25,34,36]. We have included Stevens et al. [30] in this category, which used intersectionality for both design and analysis. The main value of intersectionality in these studies was to provide the appropriate mechanisms to culturally adapt interventions, in order to cover the intersection between different types of health inequalities. In these studies, intersectionality was specifically used to address social factors related to ethnicity and gender [25,30,36], and to a lesser extent socioeconomic position and LGBTQI+ [34], within intervention design. In general, these studies applied key tenets from intersectionality frameworks with a focus on structural factors and power dynamics. 

Liu et al. interviewed 26 health researchers and promoters to explore their views on adapting interventions for ethnic minority women [25]. Researchers interviewed in this study were clear that intersectionality was helpful in understanding the combined influence of ethnicity and gender among other factors in health promotion interventions. They highlight that intersectionality, as well as the concepts of representation and contextual experiences, are all useful in understanding how adapting interventions works in practice. Bounds et al. [34] and Kelly and Pich [36] both used intersectionality frameworks to acknowledge and address issues of power and discrimination, in line with the key tenets of intersectionality. Bounds et al. [34] aimed to adapt an intervention for newly homeless youth to be used to reduce risk factors for sexual exploitation. Participants recognised the importance of acknowledging experiences of structural violence, while the authors concluded that focus groups and intersectionality are useful in considering the unique issues of disempowered youth. Kelly and Pich [36] aimed to reduce post-traumatic stress disorder and improve quality of life, social support and self-efficacy among Latinas who experienced intimate partner violence. The authors integrated both biomedical and intersectional approaches throughout their study, adapting a family intervention to reduce risk factors for sexual exploitation and acknowledging issues of power and invisibility. They concluded that that interventions helped to improve mental health related symptoms, with an intersectional approach being key to this, although there were limited impacts on other outcomes. 

The only study which used intersectionality to design and analyse their intervention was Stevens et al. [30]. This study used intersectionality to assess the effectiveness of a coordinated perinatal mental health care model focused on socially disadvantaged ethnic minority women. They use the framework to conceptualise the ‘vulnerability’ of these women as influenced by a range of interacting structural factors. They found similar treatment outcomes among ethnic minorities as in White patients and concluded that their treatment model based on intersectionality has promise to reduce inequalities in this area, over and above approaches without an intersectionality framework. 

## 4. Discussion

This scoping review aimed to explore the use of intersectionality in the design and/or assessment of interventional health research. Our findings suggest that intersectionality frameworks are not yet explicitly used in this body of research, as we identified only 12 studies in total. In the studies we identified, the axes of inequality gender, ethnicity and socio-economic position were commonly assessed, but there was less attention paid to other markers of inequality. In our included studies, intersectionality frameworks were used to pose questions about intervention effectiveness and interpret differences in outcomes, and were recommended for use in tailoring and delivering future treatments. We found that intersectionality frameworks were more commonly used to assess the impacts of pre-existing interventions, rather than to use these frameworks to develop new methods of tackling health inequalities. 

To the best of our knowledge, this is the first review studying the use of intersectionality frameworks within interventional health research. Some previous research has explored the association between intersectionality and health through theoretical, qualitative and observational studies, without considering health interventions specifically [14]. One previous review analysed evidence on the use of intersectionality in health by mapping its presence [14]. This review pointed to significant room for improvement in explicitly connecting research methods and reporting to intersectionality frameworks in studies and focusing more on interventions. Our scoping review builds on this work and the fact that we found so few studies suggests there is still some way to go before intersectionality is comprehensively incorporated into interventional research. 

Other work by Harari and Lee has identified limitations in quantitative research using intersectionality, including the prioritisation of certain groups and not others, and a lack of consideration of underlying processes [37]. While we found only a limited number of studies, those quantitative studies which we did identify chime with Harari and Lee’s assessments. Promisingly, however, there was also evidence that the included studies had taken care to consider diverse identities and drivers of inequality, including stigma, power dynamics and identity. However, some characteristics were included more commonly than others: we found ethnicity and gender to be the most commonly included intersections, but a comparative lack of consideration of LGBTQi+ and disability. This, perhaps, is unsurprising given that intersectionality was borne out of an interest specifically in the position of Black women, and that gender and ethnicity are common demographic characteristics collected in intervention research [5,38]. 

Researchers have warned against intersectionality work sliding into ‘oppression Olympics’ [39], in which we try to determine which characteristic is most important for a health outcome instead of appreciating the nuance and complexity of people’s intersectional positions. Our finding that gender and ethnicity were the most commonly included characteristics, while expected given the recognition of these as key dimensions of inequality and thus greater data availability, reflects the debates around differentiating categories within intersectionality [40]. There are three approaches to this: intra-categorical approaches that focus on complexity of experience within a particular social position or intersection, inter-categorical approaches that focus on heterogeneity across a range of intersections and anti-categorical approaches that critique rigid social categorization itself [14]. There is an argument that interventional research needs to categorise to some extent to determine the success of the strategy, with gender and ethnicity being core categories by which researchers want to understand intervention effects. Interventional research has therefore mostly used inter-categorical approaches, working within a traditionally positivist paradigm [15]. To begin to address this challenge and to uncover inequalities in a more nuanced way, there is a need for innovative and mixed methods that can explore the richness and detail of people’s experiences while also conducting intervention effect comparisons. 

The studies included used a range of methods to employ intersectionality, drawing on different concepts dependent on their methods. Although intersectionality is increasingly highlighted as a promising framework for public health research, there is still uncertainty and challenge about how to operationalise it methodologically [3]. It is therefore unclear how to determine whether intersectionality was appropriately applied to the different methods used by the included studies. What we can say is that all the included studies used intersectionality as an analytic framework, rather than approaching the presence or absence of intersectionality as a testable hypothesis [41]. This finding is positive, given calls for intersectionality to be used analytically, rather than purely descriptively. Regarding topic area, we found that mental health accounted for half of the included studies, which could be related to the burgeoning field of research that has identified social inequalities in mental health across multiple dimensions of inequality, such as socioeconomic position, gender and sexual orientation, among others [42]. Further, within mental health research, there has been increasing focus on how these dimensions of inequality may need to be incorporated into analysis to identify complex and potentially unexpected patterns in the distribution of health [42]. Finally, mental health research is well suited to intersectionality, as the people and groups occupying positions of multiple disadvantages may well experience identity issues, stigma, discrimination and disempowerment.

We found limited evidence on the explicit use of intersectionality frameworks to support the development of interventions. Those studies which did, however, had a strong recognition of the issues of power, structural dynamics and discrimination, and they considered these in intervention development. There is an inherent complexity in addressing these issues, which may explain in part why we found a limited number of studies. Furthermore, some of these components have been recognised as useful elements to tackle inequalities without being mentioned as part of any intersectionality framework [43]. Hence, rather than being underrepresented, intersectionality might be to some extent underreported, since many of its main components may be currently used in health interventions, even though they are not framed as intersectionality in any theoretical background. 

The benefits of incorporating intersectionality into public health research are well-established in theory and have been championed by scholars over the past few decades [5,10,11,14]. What our scoping review intended to contribute was evidence as to whether intersectionality is being actively used by researchers in the development and evaluation of interventions. What we found was limited use of intersectionality within interventional research, perhaps as a result of some of the points we have discussed. There is motivation from many within the public health community to adopt intersectionality as a way of reducing health inequalities but translating it into methodological approaches remains a challenge. While our small sample size limits what we can recommend in terms of intersectionality and intervention research, we would suggest that incorporating intersectionality in the design of interventions (not as a post-intervention analysis), would strengthen the equity focus. As fewer studies used intersectionality in this way, our review conveys that there is still some way to go before this becomes more commonplace. We would direct researchers to resources such as Hankivsky et al.’s [44] primer and Heard et al.’s paper [15], which both provide case study examples of innovation using intersectionality frameworks in public health research. Additionally, the ‘For-Equity’ website also provides tools and resources related to intersecting inequalities to enable researchers to think about this when developing and evaluating interventions [45]. 

### Strengths and Limitations 

This review presents a timely review of research on the use of intersectionality in interventional health research. It identifies that intersectionality is not yet commonly used in this body of research. The reduction of health inequalities is a core component of public health and has been since its inception, and so this is a highly relevant topic within public health research. Our most recent search was undertaken in June 2021, so there may be relevant studies missing from our analysis. Staff changes affected our capacity to update the searches. We used a broad, pre-defined comprehensive search strategy using two of the main biomedical databases, and the citations of identified key papers were also screened. Nonetheless, the main limitation of this review is that we only included papers explicitly mentioning intersectionality or related terms. We did pilot searches in an attempt to identify papers which used key tenets of intersectionality. This, however, was not feasible, as almost all papers would have to be read at full text stage in order to assess if they had used intersectional ideas or those of interacting identities in the consideration of their results. This means that we may have missed studies which did not use the term explicitly, but which used strategies in line with intersectionality frameworks. As such, our results likely constitute an underestimate of the extent of use of intersectionality frameworks in interventional health research.

A further limitation of our scoping review is that we limited our inclusion criteria to high income countries only. This means our findings are non-transferrable to low-and-middle income countries. There is work that explores intersectionality use in research in low-and-middle income countries [23], and also a review that does not separate in this way [24]. We would suggest that future work seeks to compare these two intervention contexts, as it may have implications for the extent and ways intersectionality is implemented. 

Our scoping review intended to map out the extent to which intersectionality is being used in interventional health research and highlight some examples of its use. An extension of this work would assess whether and how intersectionality impacts on the quality of the interventional health research comparative to the use of other theoretical and analytical frameworks. Finally, as this was a scoping review focused on exploring the use of intersectionality, we did not conduct formal critical appraisal of included studies.

## 5. Conclusions

In conclusion, this scoping review has revealed there is a lack of evidence on the use of theoretical and analytical intersectionality frameworks when designing and analysing public health interventions, with only a small number of studies identified. Where intersectionality was adopted, frameworks were used to pose questions about intervention effectiveness or interpret differences in outcomes, rather than in the design or implementation of interventions. Therefore, intersectionality within this small sample was primarily used as an analytical framework. Public health research is increasingly highlighting the value of intersectionality frameworks for attempting to reduce inequalities. The lack of studies in our review suggests that theoretical and methodological advancements need to be made in order to enhance engagement with intersectionality as part of the health intervention development and assessment cycle. 

## Figures and Tables

**Table 1:** Summary of studies features.

	*n* = 12	%
**Country**		
USA	6	50
UK	4	33.33
Canada	1	8.33
Several countries	1	8.33
**Year**		
2021	2	16.66
2020	3	25
2019	3	25
2018	1	8.33
2017	0	0
2016	1	8.33
2015	1	8.33
2014	1	8.33
**Research design**		
Qualitative	6	50
Quantitative	3	25
Mixed-method	3	25
**Intersectionality use**		
Analysis	8	66.66
Design	3	25
Design/Analysis	1	8.33

**Table 2:** Study characteristics and intersections.

Author(s)	Country	Health Topic	Sample Size	Intersectionality Use	Inequalities					
					SES	Ethnicity	Gender	LGBTQ+	Disability	Other(s)
Gleeson et al. (2020) QUAL [26]	UK	Alcohol misuse	17	Analysis						NA
Liu et al. (2016), QUAL [25]	Various	Health promotion (Various)	37	Design						Age
Lloyd et al. (2021) QUAL [27]	UK	Mental health	18	Analysis						Age
Medina-Perucha et al. (2019) QUAL [28]	UK	Drug abuse	20	Analysis						Various
Wilkinson and Ortega-Alcázar (2019) QUAL [29]	UK	Housing	40	Analysis						NA
Stevens et al. (2018) QUAL [30]	USA	Mental health (perinatal)	82	Design /Analysis						NA
Morrow et al. (2020) MIXED [31]	Canada	Mental health	94	Analysis						Age
Potter et al. (2021) QUANT [32]	USA	Smoking cessation	344	Analysis						NA
Kivlighan et al. (2019) MIXED [33]	USA	Mental health	415	Analysis						NA
Bounds et al. (2020) MIXED [34]	USA	Risk for sexual exploitation	40	Design						NA
David et al. (2015) QUANT [35]	USA	Mental health	300	Analysis						NA
Kelly and Pich (2014) QUANT [36]	USA	Mental health	27	Design						NA
					10	11	11	4	1	4

## Data Availability

All data generated or analysed during this study are included in this published article.

## Appendix A

**Table A1:** Search strategies.

		Search Strategy–Medline via Web of Science. Date: 11 May 2021	
	Blocks	Search terms	Results
# 7		#6 AND #1	1455
		Indexes = MEDLINE Timespan = All years	
# 6		#5 OR #4 OR #3 OR #2	14,113,133
		Indexes = MEDLINE Timespan = All years	
# 5	Interventions	TS = ((Public Health Practices) OR (Community health services) OR (health care rationing) OR (Healthy People Program) OR (capacity building) OR (health facilities) OR (health personnel) OR (health NEAR/2 promotion) OR (health services) OR (health care reform) OR (health plan implementation) OR (health planning technical assistance) OR (health NEAR priorities) OR (health resources) OR (national health programs) OR ((regional OR local) NEAR (public health)) OR (Preventive Health Services) OR (health NEAR education))	1,332,906
		Indexes = MEDLINE Timespan = All years	
# 4	Interventions	TS = ((intervention* OR program* OR strateg* OR quasi-experimental OR evaluat* OR evidence OR assessment OR effectiveness OR ‘health survey’ OR trial OR utilization OR access*) OR (quasi-experimental OR random$ OR ‘health survey’ OR “longitudinal study”) OR (comparative OR control* OR prospective OR evaluation OR blind* OR effective*))	13,749,807
		Indexes = MEDLINE Timespan = All years	
# 3	Interventions	MH = (Public Health Practices OR Community health services OR health care rationing OR Healthy People Program OR capacity building OR health facilities OR health personnel OR health promotion OR health services OR health care reform OR health plan implementation OR health planning technical assistance OR health priorities OR health resources OR national health programs OR regional health planning OR Preventive Health Services OR health education)	352,501
		Indexes = MEDLINE Timespan = All years	
# 2	Interventions	MH = (clinical trials OR feasibility studies OR intervention studies OR comparative studies OR evaluation studies OR Evidence-Based Practice)	83,140
		Indexes = MEDLINE Timespan = All years	
# 1	Intersectionality	TS = (intersectional*)	2099
		Indexes = MEDLINE Timespan = All years	

# = number. * = truncation of wildcard operators.

Search Strategy–Scopus. Date: 11 May 2021

((TITLE-ABS-KEY (intersectional*)) AND (TITLE-ABS-KEY (((intervention* OR program* OR strateg* OR quasi-experimental OR evaluat* OR evidence OR assessment OR effectiveness OR ‘health AND survey’ OR trial OR utilization OR access*) OR (quasi-experimental OR random$ OR ‘health AND survey’ OR “longitudinal study”) OR (comparative OR control* OR prospective OR evaluation OR blind* OR effective*))))) AND (TITLE-ABS-KEY (((health AND inequalit*) OR (health AND inequit*) OR (social AND depriv*) OR (social AND disadvantage*) OR (education* AND status) OR (equity) OR (“in need”) OR (poverty) OR (low-income) OR (underserved) OR (inequit*) OR (inequality*) OR (disadvantage*) OR (disparit*) OR (discrimination))))

## Appendix B

**Table A2:** Total number of references: 1113.

Author(s)	Country	Target Population	Health Issue(s)	Intervention	Intersectionality Framework	Summary of Results
Gleeson, Herring, and Bayley (2020) [26]	UK (London-based)	Polish migrants and professionals	Alcohol misuse	Health promotion	The analysis has attempted to incorporate the experiences of migration, gender, alcohol use and social class to give a broad understanding of pathways into, through and beyond alcohol treatment.	The findings suggest a need for services to address the unique service needs of Polish (and potentially other migrant) women, including additional social stigma, social attitudes toward women within minority communities surrounding substance use and challenges to engaging with treatment. The professionals highlighted Polish migrant women’s likelihood of being dependent on a male partner both in terms of financial security and access to social networks. The multiple references from professionals relating to the interaction between alcohol use and domestic violence for this group of women also suggests a need for treatment services to be aware of the additional negative experiences of women and seek ways to ensure they are addressed within treatment programmes.
Liu et al. (2016), [25]	USA, UK, Australia, New Zealand and Norway	Researchers	Smoking cessation, increasing physical activity and healthy eating	Health promotion	An intersectional perspective in this study highlights both the mutually constitutive positive and negative effects these factors have on the social identities of members of ethnic minority populations, and their health practices and outcomes.	Findings include (i) the intersections of ethnicity and demographic variables such as age and gender highlight the different ways in which people interact, interpret and participate in adapted interventions; (ii) the representational elements of ethnicity such as ancestry or religion are more complexly lived than they are defined in adapted interventions; (iii) the contextual experiences surrounding ethnicity considerations shape the receptivity, durability and continuity of adapted interventions.
Lloyd, Rimes, and Hambrook (2021) [27]	UK (London-based)	Service users who had previously attended and completed the LGBQ Wellbeing Group were collated.	Mental health (anxiety and depression)	Mental health	Intersectional approaches are situated in the understanding that individual identities are built on multiple different layers relating to different aspects of our socially defined selves, such as gender, gender identity, sexuality, race/ethnicity, social class, etc.	Respondents reported that they found the CBT frame of the group useful, with the LGBQ focus experienced as particularly beneficial, often enhancing engagement with CBT concepts and tools. In addition to generic elements of group therapy that some found difficult, others reported that intragroup diversity, such as generational differences, could lead to a reduced sense of connection. Several suggestions for group improvement were made, including incorporating more diverse perspectives and examples in session content and focusing more on issues relating to intersectionality.
Medina-Perucha et al. (2019) [28]	UK	Women, over 18 years of age, and on/having received opioid substitution treatment	Drug abuse	Health promotion	Drug use-related stigma often overlaps with stigma associated with other interdependent social categories. Personal and social identity can actually be understood as multidimensional rather than the unidimensional product of a combination of personal attributes and belonging to certain social groups. An individual can experience multiple overlapping stigmas (intersectional stigma) that refer to associations between social identities and structural inequities.	Women’s narratives highlighted the intersection of stigma associated with distinct elements of women’s identities: (1) female gender, (2) drug use, (3) transactional sex, (4) homelessness and (5) sexual health status. Intersectionality theory and social identity theory are used to explain sexual health risks and disengagement from (sexual) health services among women on opioid substitution treatment (WOST). Intersectional stigma was related to a lack of female and male condom use and a lack of access to (sexual) health services.
Wilkinson and Ortega-Alcázar (2019) [29]	UK (England and Wales)	Single people, without dependents, aged between 18 and 24	Housing, Physical safety and harassment, mental health and isolation	Housing	An intersectional framework attempts to take into consideration the ways in which people are multiply marginalized by different, but interlinked, social structures: such as class, racism, sexism, homophobia and ableism.	The analysis focuses on two key themes: physical safety and violence, followed by mental health and isolation. Ultimately, the paper examines whether housing welfare reform in Britain has resulted in placing already vulnerable people into potentially dangerous and unhealthy housing situations.
Stevens et al. (2018) [30]	USA	Pregnant and postpartum women	Perinatal mental health	Mental health treatment	Intersectionality attends to the interactive relationships among social factors such as race, ethnicity, education, partner status, income, geography and other factors that play a key role in perinatal mental health and adjustment to parenting. Viewed through this lens, perinatal women’s experiences as “socially vulnerable” are influenced by the complex interweaving of numerous possible factors.	Results showed high treatment engagement and effectiveness, with 65.9% of participants demonstrating reliable improvement in symptoms. African American and Hispanic/Latina patients had similar treatment outcomes compared to White patients, despite facing greater socio-economic disadvantages. Findings indicate that the treatment model may be a promising approach to reducing perinatal mental health disparities.
Morrow et al. (2020) [31]	Canada	Participants were Asian men living with or affected by mental illness and community leaders interested in stigma reduction and advocacy.	Mental health	Mental health	As an analytic framework, intersectionality helps to clarify the complex interactions between age and ableism, colonization and white supremacy, heteronormativity and hegemonic masculinity, xenophobia and neoliberalism. This paper use intersectionality to explore Asian men’s experiences of stigma and mental illness specifically to tease out the ways in which stigma of mental illness among Asian men is mediated by age, immigration experiences, sexual and gender identities, racism and racialization processes, normative expectations about masculinity and material inequality.	The data collected pre- and post-interventions revealed that men understand and experience stigma as inextricably linked to social location, specifically age, race, masculinity, ethnicity and time of migration. Our analysis also revealed that mental health stigma cannot be understood in isolation from other social and structural barriers. The application of intersectional frameworks must figure prominently in psychological research and in public health policies that seek to reduce mental health stigma in racialized communities.
Potter, Lam, Cinciripini, and Wetter (2021) [32]	USA	Participants were 424 male and female adult smokers	Smoking cessation	Health promotion	An intersectionality framework is useful for understanding how the interplay between multiple marginalized sociodemographic attributes may shape health inequities. This work highlights the importance of moving beyond prioritizing one category of social status as the basis for health inequities research.	Lower household income may be related to higher risk of smoking cessation failure. There were no significant interactions among race/ethnicity, gender and income in predicting relapse. Pairwise intersectional group differences suggested some groups may be at higher risk of relapse. Number of marginalized sociodemographic attributes did not predict relapse.
Kivlighan et al. (2019) [33]	USA	Clients who received one treatment episode of individual counselling	Mental health	Mental health	Intersectionality theory seeks to understand and promote the inseparable intersection of cultural identities.	Results indicated that therapists differed in their ability to produce changes in symptom-defined psychological distress as a function of clients’ intersecting identities of race-ethnicity and gender.
Bounds, Otwell, Melendez, Karnik, and Julion (2020) [34]	USA	Four focus groups were held with mainly African American youth. The majority of the experts were female and from an ethnic/racial minority background.	Reduce risk factors for sexual exploitation	Behavioural	Intersectionality is a theoretical framework that situates multiple microlevel experiences within macrolevel systems of privilege and oppression. Authors propose to take an intersectionality approach for the intervention with homeless youth and refine the content and approach to consider the layered risks associated with their age, race/ethnicity, sexual exploitation history, sexual/gender identity and family functioning.	Results from 29 youths and 11 providers indicate that there are unique considerations that must be taken into consideration while working with youth at risk of sexual exploitation to ensure effective service delivery and/or ethical research. Emergent themes included: setting the stage by building rapport and acknowledging experiences of structural violence, protect and hold which balances youth’s need for advocacy/support with their caregivers’ need for validation/understanding and walking the safety tightrope by assessing risks and safety planning.
David, Rowe, Staeheli, and Ponce (2015) [35]	USA	Homeless women	Mental health	Mental health	The framework of intersectionality highlights the ways in which interpersonal constructs—including race, class and gender—may coincide with social structures to dynamically shape an individual’s lived experience and sense of self. An intersectionality perspective maintains that social constructs including racism, sexism and other forms of discrimination can contribute to the development of an individual’s multiple marginalized personal identities.	Authors highlight four key principles that can optimize and promote the recovery outcomes of these women: (1) peer support, (2) flexible services and resources, (3) supportive program leadership and (4) gender-sensitive services provided by women. We provide case vignettes highlighting how each of these treatment principles fosters trust and helps to create safe psychological and physical spaces for women clients.
Kelly and Pich (2014) [36]	USA	Latinas with PTSD who experienced IPV	Mental health	Mental health	An intersectional approach frames the problem as one of power inequities at multiple levels–interpersonal, institutional and societal and multiple systems (race, ethnicity, gender/class). The integration of biomedical and intersectional approaches in this study meant that both the women’s PTSD systems and intersectional invisibility were acknowledged and addressed throughout the research study.	Significant reductions in PTSD and MDD and increased self-efficacy were sustained 6 months post-intervention. Culturally relevant mental health IPV interventions can be feasible and appropriate in women across ethnic groups. However, there were not significant impact on other outcomes such as quality of life.
